# Surface Functionalization of Poly(l-lactide-*co*-glycolide) Membranes with RGD-Grafted Poly(2-oxazoline) for Periodontal Tissue Engineering

**DOI:** 10.3390/jfb13010004

**Published:** 2022-01-07

**Authors:** Anna M. Tryba, Małgorzata Krok-Borkowicz, Michał Kula, Natalia Piergies, Mateusz Marzec, Erik Wegener, Justyna Frączyk, Rainer Jordan, Beata Kolesińska, Dieter Scharnweber, Czesława Paluszkiewicz, Elżbieta Pamuła

**Affiliations:** 1 Department of Biomaterials and Composites, Faculty of Materials Science and Ceramics, AGH University of Science and Technology, Al. Mickiewicza 30, 30-059 Kraków, Poland; amtryba@agh.edu.pl (A.M.T.); krok@agh.edu.pl (M.K.-B.); kula.michal96@gmail.com (M.K.); 2 Institute of Nuclear Physics, Polish Academy of Sciences, ul. Radzikowskiego 152, 31-342 Kraków, Poland; natalia.piergies@ifj.edu.pl (N.P.); Czeslawa.Paluszkiewicz@ifj.edu.pl (C.P.); 3 Academic Centre for Materials and Nanotechnology, AGH University of Science and Technology, Al. Mickiewicza 30, 30-059 Kraków, Poland; marzecm@agh.edu.pl; 4 Faculty of Chemistry and Food Chemistry, Technische Universität Dresden, Mommsenstr, 401069 Dresden, Germany; erik.wegener@mailbox.tu-dresden.de (E.W.); Rainer.Jordan@tu-dresden.de (R.J.); 5 Faculty of Chemistry, Institute of Organic Chemistry, Łódź University of Technology, ul. Żeromskiego 116, 90-924 Łódź, Poland; justyna.fraczyk@p.lodz.pl (J.F.); kolesins@p.lodz.pl (B.K.); 6 Max Bergmann Center of Biomaterials, Technische Universität Dresden, Budapester Str. 27, 01069 Dresden, Germany; Dieter.Scharnweber@tu-dresden.de

**Keywords:** poly(l-lactide-*co*-glycolide), poly(ethylene glycol), poly(2-oxazoline), RGD sequences, phase separation, periodontology, osteoblast-like cells, guided tissue regeneration (GTR), bone tissue engineering

## Abstract

Bone tissue defects resulting from periodontal disease are often treated using guided tissue regeneration (GTR). The barrier membranes utilized here should prevent soft tissue infiltration into the bony defect and simultaneously support bone regeneration. In this study, we designed a degradable poly(l-lactide-*co*-glycolide) (PLGA) membrane that was surface-modified with cell adhesive arginine-glycine-aspartic acid (RGD) motifs. For a novel method of membrane manufacture, the RGD motifs were coupled with the non-ionic amphiphilic polymer poly(2-oxazoline) (POx). The RGD-containing membranes were then prepared by solvent casting of PLGA, POx coupled with RGD (POx_RGD), and poly(ethylene glycol) (PEG) solution in methylene chloride (DCM), followed by DCM evaporation and PEG leaching. Successful coupling of RGD to POx was confirmed spectroscopically by Raman, Fourier transform infrared in attenuated reflection mode (FTIR-ATR), and X-ray photoelectron (XPS) spectroscopy, while successful immobilization of POx_RGD on the membrane surface was confirmed by XPS and FTIR-ATR. The resulting membranes had an asymmetric microstructure, as shown by scanning electron microscopy (SEM), where the glass-cured surface was more porous and had a higher surface area then the air-cured surface. The higher porosity should support bone tissue regeneration, while the air-cured side is more suited to preventing soft tissue infiltration. The behavior of osteoblast-like cells on PLGA membranes modified with POx_RGD was compared to cell behavior on PLGA foil, non-modified PLGA membranes, or PLGA membranes modified only with POx. For this, MG-63 cells were cultured for 4, 24, and 96 h on the membranes and analyzed by metabolic activity tests, live/dead staining, and fluorescent staining of actin fibers. The results showed bone cell adhesion, proliferation, and viability to be the highest on membranes modified with POx_RGD, making them possible candidates for GTR applications in periodontology and in bone tissue engineering.

## 1. Introduction

Periodontal disease (PD) is a common chronic inflammatory oral disorder caused by periodontal pathogens present in the oral cavity. The clinical pathological characteristic of PD is the progressive and irreversible destruction of soft and hard periodontal tissues. PD is the main cause of tooth loss in adults and other diseases that endanger human oral and overall health [1].

PD treatment aims to control inflammation and prevent the further development of periodontal pathology, while also to restoring the structure and function of lost periodontal tissue. Periodontal tissue regeneration is a long-standing and difficult challenge. Conservative treatment can inhibit PD’s progression; however, it does not restore the tooth support from bone or connective tissue.

Guided tissue regeneration (GTR) is a surgical procedure that specifically aims to regenerate periodontal tissues when the disease is advanced, and it may override some of the limitations of conventional therapy [2]. GTR is based on the principle of guiding the proliferation of restorative periodontal tissues following periodontal surgery. Nyman et al. were the first to introduce the concept of GTR using subgingival barrier membranes [3]. The therapeutic achievements obtained by using membranes in GTR treatment include the prevention of apical migration of epithelial cells, promotion of progenitor cell growth, and exclusion of gingival connective tissues [4]. Therefore, it is beneficial if the membranes are of graded porosity in the cross section; that is, they are less porous on the surface intended to be in contact with the gum, while being more porous on the surface contacting the bone tissue defect to promote osteogenic cell adhesion, proliferation, and differentiation, and thus bone tissue restoration [5,6].

Specially designed non-absorbable or absorbable membranes play a key role in periodontal GTR [7]. Non-absorbable membranes are made of porous expanded polytetrafluoroethylene (e.g., ePTFE from Gore-Tex) or dense PTFE. More recently, textured dense PTFE membranes and those reinforced with titanium mesh have been introduced to the market. However, the main disadvantage of non-absorbable membranes is the second traumatic surgery needed to remove them from the treated place after ca. 6 months [8].

Absorbable membranes, of natural or synthetic origin, do not require removal after tissue regeneration, which reduces patient discomfort and eliminates surgery-related complications. Natural, absorbable collagen membranes (e.g., Bio-Gide from Geistlich), are often regarded as a gold standard due to their biocompatibility and improvement of healing [9]. However, their animal origin and possible transmission of bovine spongiform encephalopathy (BSE) are of concern [10].

Synthetic absorbable barrier membranes can be made of aliphatic polyesters, e.g., poly(glycolic acid) (PGA), poly(lactic acid) (PLA), and their copolymer poly(lactide-*co*-glycolide) (PLGA). These polymers degrade by hydrolysis into products that are metabolized to CO_2_ and H_2_O through the citric acid/Kreb’s cycle. The degradation rate depends on the properties of the surrounding environment (pH, presence of enzymes, and mechanical loads), and the structure and properties of the materials (chemical composition, degree of cross-linking, crystallinity) [11]. A double-layered absorbable GTR membrane (Guidor) made of poly(lactic acid) and a citric acid ester (acetyl tributyl citrate) was the first commercially available membrane [9]. In our previous studies, we developed a method for the manufacture of PLGA membranes with graded porosity through phase separation in a PLGA–poly(ethylene glycol) (PEG)–dichloromethane (DCM) system, followed by the evaporation of DCM and leaching of PEG [12,13].

These PLGA membranes also have potential to help PD treatment due to their appropriate mechanical properties [12]; however, the lack of cell adhesion motifs on their surface does not support the early adhesion of bone and osteogenic cells, which is important in the restoration of bone tissue in the periodontal lesion. Recently, in the literature, a great deal of attention has been paid to the development of advanced hybrid and surface-functionalized biomaterials with tailored properties, including those containing integrated functionalities [14,15,16]. Thus, our idea was to produce PLGA membranes with cell adhesion molecules exposed on their surface. Arginine-glycine-aspartic acid (RGD) polypeptides are known for their strong interaction with the αvβ5 and αvβ3 integrins and have been used in the modification of different biomaterials to enhance cell adhesion [17,18,19,20,21].

To this end, we coupled RGD to a non-ionic amphiphilic polymer –poly(2-oxazoline) (POx), thus forming POx_RGD, and developed an original membrane manufacturing method according to the phase separation/porogen (PEG) leaching approach [22]. We designed a system in which POx_RGD would be preferentially accumulated at the interface of PLGA/PEG solution in the nonpolar solvent, with the RGD motifs exposed toward hydrophilic PEG. We hypothesized that after solvent evaporation and PEG leaching, POx_RGD would be preferentially immobilized on the PLGA surface because of hydrophobic interactions with the hydrophobic domain of POx. The aim of this study was to confirm that the modification of the PLGA membrane with POx_RGD was successful and improved bone cell adhesion, viability, and proliferation, which are of key importance in GTR in periodontology and bone tissue engineering.

## 2. Materials and Methods

### 2.1. Materials and Chemicals

**Figure 1:**
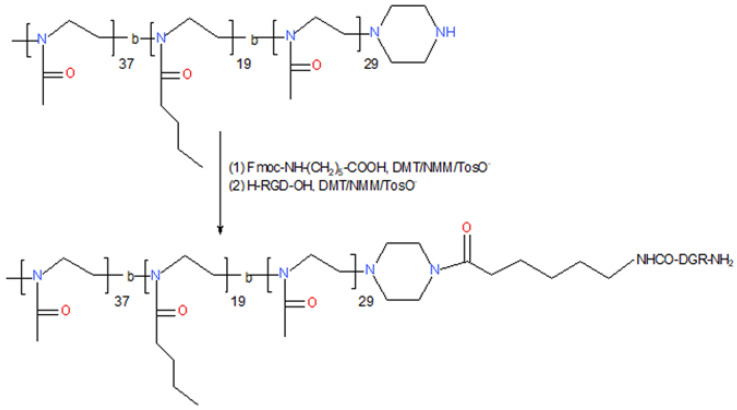
Modification method of POx (first line) with RGD (POx_RGD, second line).

The membranes were produced using poly(l-lactide-*co*-glycolide) (PLGA, 85:15 molar ratio of l-lactide to glycolide units, Mn = 100 kDa, d = 1.9), synthesized with the use of zirconium(IV) acetylacetonate initiator and kindly provided by Prof. P. Dobrzyński from the Center of Polymer and Carbon Materials, Polish Academy of Sciences in Zabrze, Poland. As a solvent dichloromethane (DCM) and as a pore former PEG (Mn = 400 Da), both from Sigma-Aldrich, were used. As an amphiphilic polyoxazoline molecule, (POx) poly(2-methyl-2-oxazoline-b-2-butyl-2-oxazoline-b-2-methyl-2-oxazoline), to be more precise methyl-P[MeOx37-b-BuOx_23_-b-MeOx_37_-piperidine(P2–P2) (Mn = 8 kDa, d = 1.14), synthesized according to the previously published procedure [23] was bought from Technische Universität Dresden, Germany (POx structure is shown in Figure 1, first line). Moreover, POx was modified with an RGD-derivative with 6-aminohexanoic acid. The coupling reaction took place exclusively at the NH group of the piperazine ring. For coupling, the RGD-6-aminocaproic acid derivative was used, which was coupled to the NH group by means of the free carboxylic acid of 6-aminocaproic acid. In the first step, Fmoc-protected 6-aminocaproic acid was attached to POx by using reagent 4-(4,6-dimethoxy-1,3,5-triazin-2-yl)-4-methylmorpholinium toluene-4-sulfonate (DMT/NMM/ TosO^−^). After removal of the Fmoc group with a 25% solution of piperidine in N,N-dimethylformamide (DMF) (standard SPPS procedure), the H-RGD-OH peptide was attached to POx-CONH-(CH_2_)_5_-NH_2_. For the coupling, DMT/NMM/TosO^−^ was used, the efficiency of which was also checked in the coupling of the fragments. As a result, POx_RGD was obtained (Figure 1, second line).

### 2.2. Membrane Manufacturing Method

**Figure 2:**
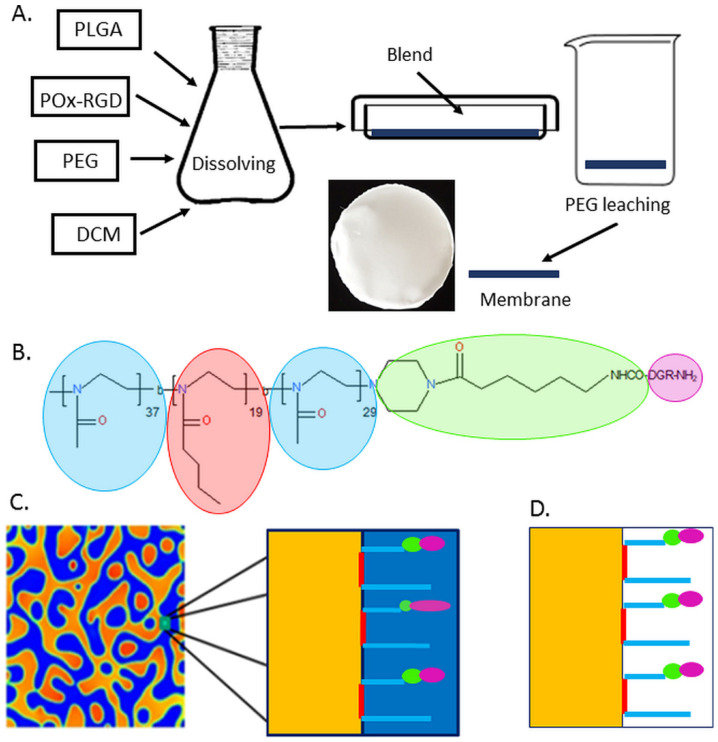
(**A**) Scheme of manufacturing procedure of PLGA membrane with Pox_RGD. (**B**) Chemical structure of POx_RGD with characteristic domains highlighted in cyan (2-metyl-2-oxazoline), pink (2-butyl-2-oxazoline), green (6-aminocaproic acid coupled to the piperazine ring), and purple (RGD). (**C**) Scheme of phase separation in the PLGA–PEG–POx_RGD system and immobilization of POx_RGD at the interface of PLGA (yellow colour) and PEG (blue colour). (**D)** Surface-modified PLGA membrane with POx_RGD after PEG leaching.

To obtain the membranes, PLGA and PEG were co-dissolved in DCM at a concentration of 10% wt./vol. and 1 wt. % POx_RGD, in respect to PLGA, was added. The membranes were obtained by solvent casting on glass Petri dishes, followed by air drying for 24 h and vacuum drying for 24 h. After that time, the PLGA/PEG/POx_RGD blends were washed in purified water (UHQ-water, Pure Lab, UK) for 5 days; the water was changed every 30 min during day 1 and every 2 h from days 2 to 5 (Figure 2A), resulting in the preparation of the membranes PLGA_POx_RGD. As references PLGA foils, PLGA membranes without additives, and PLGA membranes with POx were prepared. Figure 2B shows the chemical structure of POx_RGD with characteristic domains highlighted in cyan (2-metyl-2-oxazoline), pink (2-butyl-2-oxazoline), green (6-aminocaproic acid coupled to the piperazine ring), and purple (RGD). Figure 2C shows a scheme of the phase separation in the PLGA–PEG–POx_RGD system and deposition of POx_RGD at the interface of PLGA (yellow colour) and PEG (blue colour). This resulted in a surface-modified PLGA membrane with POx_RGD after PEG leaching (Figure 2D).

### 2.3. Evaluation Methods

#### 2.3.1. Raman Spectroscopy

The Raman spectroscopy analyses were performed using an inVia Renishaw spectrometer system (Renishaw, Wotton-under-Edge, UK) working with a Leica confocal microscope and the 100× magnification objective. A laser line at 785 nm with 100% of the laser power as an excitation source and the thermoelectrically cooled CCD detector were applied. The spectra were acquired with one scan and 30 s of integration time in the range of 100 and 3200 cm^−1^. The spectral resolution was ca. 1 cm^−1^.

#### 2.3.2. FTIR-ATR

Fourier transform infrared (FTIR) spectroscopy in attenuated total reflection (ATR) mode spectra were collected using a Nicolet iZ10 (Thermo Scientific, Waltham, MA, USA) spectrometer with a Zn attenuated total reflection (ATR) crystal and resolution of 4 cm^−1^. The number of scans was 64.

#### 2.3.3. X-ray Photoelectron Spectroscopy

The XPS analyses were carried out in a PHI VersaProbe II Scanning XPS system using monochromatic Al Kα (1486.6 eV) X-rays focused to a 100 µm spot and scanned over an area of 400 µm × 400 µm. The photoelectron take-off angle was 45° and the pass energy in the analyzer was set to 117.50 eV (0.5 eV step) for survey scans and 46.95 eV (0.5 eV step) to obtain high energy resolution spectra for the C_1s_, O_1s_, N_1s_, S_2p,_ and Si_2p_ regions. A dual beam charge compensation with 7 eV Ar^+^ ions and 1 eV electrons was used to maintain a constant sample surface potential regardless of the sample conductivity. All XPS spectra were charge referenced to the unfunctionalized, saturated carbon (C–C(H)) C_1s_ peak at 284.8 eV. The operating pressure in the analytical chamber was less than 3 × 10^−9^ mbar. The deconvolution of the spectra was carried out using PHI MultiPak software (v.9.9.0.8, ULVAC-PHI, Chigasaki, Japan). The spectrum background was subtracted using the Shirley method.

#### 2.3.4. Scanning Electron Microscopy

The microstructure of the samples was observed with scanning electron microscopy (SEM, Nova Nano SEM 200, Hillsboro, OR, USA) after sputtering the samples with a 10 nm carbon layer to make them conductive. To study material cross-sections, the samples were immersed in liquid nitrogen prior to breaking.

#### 2.3.5. Wettability and Surface Free Energy

Water and diiodomethane contact angle values were measured using a drop shape analyzer (DSA25E, Krüss, Hamburg, Germany) by the evaluation of 10 individual droplets of 0.5 µL in volume. The surface free energy was calculated based on the Owens–Wendt equation, using analytical grade purified water (UHQ-water) as a polar liquid and diiodomethane from Sigma-Aldrich (St. Louis, MO, USA) as a non-polar liquid.

#### 2.3.6. In Vitro Cell Culture Tests

Cytocompatibility tests were performed on osteoblast-like MG-63 cells (European Collection of Cell Cultures, Salisbury, UK). All chemicals used in the in vitro tests were from Sigma-Aldrich (St. Louis, MO, USA) unless stated otherwise. At the beginning of the experiment, the samples were placed in the wells of 24-well plates and incubated in 70% ethanol for 20 min; after that, the samples were transferred to 24-well plates and left under a laminar hood and UV lamp overnight for sterilization. A total of 1.5 × 10^4^ cells suspended in 1 mL of cell culture medium (MEM, PAN-Biotech, Aidenbach, Germany) supplemented with 10% fetal bovine serum, 1% penicillin/streptomycin, 0.1% amino acids, and sodium pyruvate (PAN-Biotech, Germany) were poured on the samples, and the cells were cultured at 37 °C under a humidified atmosphere with 5.0% CO_2_ for 4, 24, and 96 h.

Cell viability was tested using the Alamar Blue reagent (In Vitro Toxicology Assay Kit, resazurin based, Sigma-Aldrich, St. Louis, MO, USA). The cell culture medium was carefully removed from the samples and changed to 1 mL of fresh cell culture medium containing 10% Alamar Blue. After incubation (3 h), the medium (100 μL) was transferred into a black 96-well plate for fluorescence measurement (λex = 544 and λem = 590 nm, FluoStar Omega, BMG Labtech, Ortenberg, Germany). The following formula (1) was used to calculate percentage resazurin reduction:

(1) $$\%\;rezasurin\;reduction=\frac{S_{x}-S_{control}}{S_{100\%reducted}-S_{control\;}}\;\cdot \;100\%$$

where:

*S_x_* is the fluorescence of the samples;
*S_control_* is the fluorescence of the MEM with 10% Alamar Blue reagent and without cells (0% reduction of resazurin);
*S*_100%*reduced*_ is the fluorescence of the MEM with 10% Alamar Blue reagent autoclaved at 121 °C for 15 min (100% reduction of resazurin).

Live/dead staining was carried out to observe cell attachment, spreading, and viability. The samples were washed with phosphate buffered saline (PBS), stained using calcein AM (0.1%) and propidium iodide (0.1%), and incubated for 20 min (37 °C). The cells were also stained with phalloidin and 4′,6-diamidino-2-phenylindole (DAPI) to visualize actin fibers and nuclei, respectively. The samples were washed two times in PBS and observed under an inverted microscope with an HXP 120 C metal halide (Axiovert 40, Carl Zeiss, Jena, Germany).

## 3. Results and Discussion

### 3.1. Characterization of Ingredients by Spectroscopic Methods

Prior to the manufacturing of the PLGA membranes surface-modified with POx_RGD for GTR, all the ingredients, i.e., PLGA, PEG, POx, RGD, and POx_RGD, were characterized by Raman, FTIR, and XPS spectroscopies.

#### 3.1.1. Raman Spectroscopy

**Figure 3:**
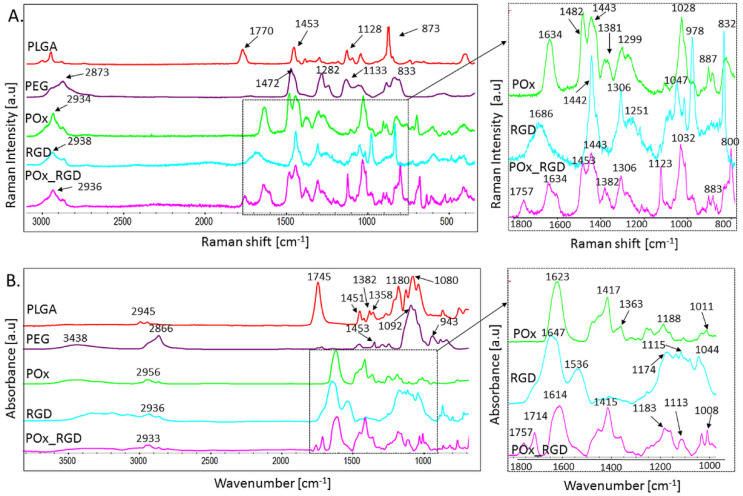
(**A**) Raman and (**B**) FTIR-ATR spectra of all ingredients used in the manufacturing of PLGA_POx_RGD membranes: PLGA, PEG, POx, RGD, and POx_RGD.

Figure 3A shows the Raman spectra and Table 1 lists the characteristic Raman bands together with their suggested assignments.

In the PLGA spectrum, a strong band at 873 cm^−1^ was present, which can be assigned to the stretching vibration of C−C=O groups. In addition, we observed bands at 1770 cm^−1^ and 1453 cm^−1^, which are due to the C=O stretching and CH bending vibrations, respectively. At 1128 cm^−1^, we observed a band attributed to C–O stretching vibrations. In the range of 2800–3000 cm^−1^, there were bands originating from the symmetric and asymmetric stretching vibrations of CH_2_ and CH_3_ groups [21,22,23].

For PEG, there were bands in the range of 2700–3000 cm^−1^, characteristic of the antisymmetric and symmetric stretching vibration of CH_2_ groups, where the band at 1472 cm^−1^ can be assigned to the symmetric stretching vibration of CH_2_–CH_2_ groups, and the band at 1282 cm^−1^ can be attributed to the CH_2_ twisting vibration. The band at 1133 cm^−1^ is attributed to C–C and C–O stretching vibrations, while the band at 833 cm^−1^ results from skeletal vibrations of the PEG molecules [24].

For POx, we observed characteristic spectral features in the range of 2800–3000 cm^−1^ (CH_2_ and CH_3_ stretching vibrations), 1634 cm^−1^ (C–N stretching) [25], 1482 and 1443 cm^−1^ (CH_2_ bending), 1381 cm^−1^ (CH_3_ symmetrical deformation/CH_2_ bending), 1299 cm^−1^ (C–N stretching), and 1028 cm^−1^ (C–C stretching) [26,27]. More assignments are shown in Table 1.

For the RGD sequences, we noticed a band in the range at 2800–3000, which can be assigned to asymmetric and symmetric CH_2_ stretching. The band at 1668 cm^−1^ is attributed to the amide I region (mainly related to the C=O stretching). The spectral features observed at 1442 cm^−1^ are responsible for sigma CH_2_ vibration, and at 1251 cm^−1^ for amide III, while the band around 1047 cm^−1^ is assigned to a beta-sheet conformation. The bands at lower shifts (lower than 1000) are assigned to H-bonding [28]. More assignments are shown in Table 1.

The Raman spectrum of POx_RGD might be regarded as the superposition of the POx and RGD spectra. We observed all the characteristic bands for POx and RGD. Interestingly, two additional bands were created at 1123 cm^−1^ (from the C–N stretching vibrations) and 1757 cm^−1^ (from the carbonyl group stretching modes), suggesting the presence of vibrations originating from the 6-aminohexanoic acid coupling agent, which was used to modify POx with RGD. The appearance of a band of strong intensity at 1123 cm^−1^, attributed to the C–N stretching vibrations [27], was not observed in the spectra of POx and RGD alone, proving the formed connection between the diazinane (piperidine) ring and the coupling agent. Such a spectral pattern was already observed by Tasal et al. [29] for the benzoxazole and piperidine rings.

The aforementioned statement is also supported by the presence of a clear shift of the Raman band at 1032 cm^−1^ for POx_RGD, assigned to the wagging HC–N/stretching C–C vibrations of the diazinane (pyridine) moiety, in comparison with the corresponding spectral feature visible in the Raman spectrum of POx at 1028 cm^−1^.

**Table 1:** The Raman bands and their assignments for the ingredients used to produce the surface-modified PLGA membranes.

Sample	Band Position (cm^−1^)	Assignments
PLGA	2800–3000	CH_2_ and CH_3_ antisymmetric and symmetric stretching [30]
	1770	C=O stretching [30,31]
	1453	CH bend [32]
	1128	C–O stretching [32]
	873	C–C=O stretching [30]
PEG	2700–3000	CH_2_ antisymmetric and symmetric stretching [24]
	1472	CH_2_–CH_2_ symmetric and antisymmetric bending [24]
	1282	CH_2_ twisting [24]
	1133	C–C and C–O stretching [24]
	833	skeletal vibrations [24]
POx	2800–3000	CH_2_/CH_3_ antisymmetric and symmetric stretching
	1634	C–N stretching [25]
	1482 and 1443	CH_2_ group plane bending [25]
	1381	CH_3_ symmetrical deformation/CH_2_ bending [26]
	1299	C–N stretching [26]
	1028	C–C stretching [26,27]
	887	CH_2_ rocking
RGD	2800–3000	CH_2_ stretching [33]
	1668	Amide I, C=O stretching [28]
	1442	CH_2_ groups plane bending [28]
	1306	C–N stretching and NH bending [28]
	1251	Amide III [28]
	1047	C–C stretching [28]
	978	C–C stretching [34]
POx_RGD	2800–3000	CH antisymmetric and symmetric stretching [28]
	1757	Carbonyl group from 6-aminohexanoic acid coupling agent stretching
	1634	C–N stretching [25]
	1481 and 1443	CH_2_ groups plane bending [25]
	1382	CH_3_ symmetrical deformation/CH_2_ bending [26,35]
	1306	C–N stretching and NH bending [28]
	1123	C–N stretching [27]
	1032	HC–N wagging/C–C stretching of diazinane (pyridine) [29]
	883	CH_2_ rocking

#### 3.1.2. FTIR Spectroscopy

Figure 3B shows the FTIR spectra characteristic of the particular ingredients used in the preparation of the membranes. In the PLGA spectrum, a strong band at 1745 cm^−1^ was present; this band can be assigned to the stretching vibration of the carbonyl groups. There were also bands between 1450 and 1360 cm^−1^, originating from CH bending vibrations, as well as bands at 1180 and 1080 cm^−1^ from C–O–C stretching vibrations. More assignments are shown in Table 2 [33,34,35,36].

In the spectrum of PEG, characteristic bands were visible at 3438 cm^−1^ and at 2866 cm^−1^ due to the stretching vibrations of the hydroxyl and CH_2_ groups, respectively. At 1453 cm^−1^, we observed a band originating from CH bending vibrations. Moreover, at 1092 cm^−1^, there was a band assigned to C–O stretching vibrations. At the lower wavenumbers in the spectral range of 840–950 cm^−1^, bands assigned to the bending vibration of CH_2_ were observed [36,37,38].

In the spectrum of POx, there was a band at 1623 cm^−1^ originating from C=O stretching vibrations of the amide group, which is characteristic of polyoxazolines [36]. Moreover, there were bands at 1417 cm^−1^ and 1363 cm^−1^, which are assigned to CH bending vibrations. There were also weak bands at 1188 cm^−1^ and 1011 cm^−1^ originating from C–N stretching vibrations [39].

When it comes to the spectrum of the RGD polypeptide, the two bands visible at 1647 cm^−1^ are associated with amide I (mainly related to the C=O stretching) vibration and at 1536 cm^−1^ are associated with amide II from the N–H bending vibrations and the C–N stretching vibrations [40]. Bands were also distinguishable at 1174, 1115, and 1044 cm^−1^, which might be attributed to the C–O and C–OH stretching vibrations [41]. More assignments are shown in Table 2.

In the FTIR spectrum of POx_RGD, all bands typical for POx were observed, and the most intensive bands originating from RGD included amide I and amide II. Interestingly, the FWHM (full width at half maximum) of the band attributed to the vibrations of the C=O bond in POx increased and its position shifted to higher wavenumbers. Moreover, two new bands at 1714 cm^−1^ and 1757 cm^−1^ appeared, which originate from the carbonyl groups created due to the coupling reaction of POx and RGD with 6-aminohexanoic acid.

**Table 2:** The FTIR bands and their assignments for the ingredients used to produce the surface-modified membranes.

Sample	Band Position (cm^−1^)	Assignments
PLGA	2945	CH stretching [36]
	1745	C=O stretching [42,43]
	1451, 1382, 1358	CH bending [44]
	1180	C–O–C stretching [43]
	1080	C–O–C stretching [36]
PEG	3438	Hydroxyl groups stretching [37,38]
	2866	CH_2_ stretching [36]
	1453	CH bending [36]
	1092	C–O stretching [36]
	943	CH_2_ bending [36]
POx	2956	CH stretching [39]
	1623	C=O stretching [36]
	1417 and 1363	CH bending [36]
	1188 and 1011	C–N stretching [39]
RGD	2936	CH stretching [40]
	1647	Amide I, C=O stretching [40]
	1536	Amide II, N–H bending, and C–N stretching [40]
	1174, 1115, 1044	C–O and C–OH stretching [41]
	869	Amide V, out of plane N–H bending [40]
POx_RGD	2933	CH stretching
	1757 and 1714	Carbonyl group from 6-aminohexanoic acid coupling agent stretching
	1614	Amide I, C=O stretching
	1415	CH bending
	1183, 1008	C–N stretching
	1113	C–O and C–OH stretching

#### 3.1.3. XPS Analysis

**Figure 4:**
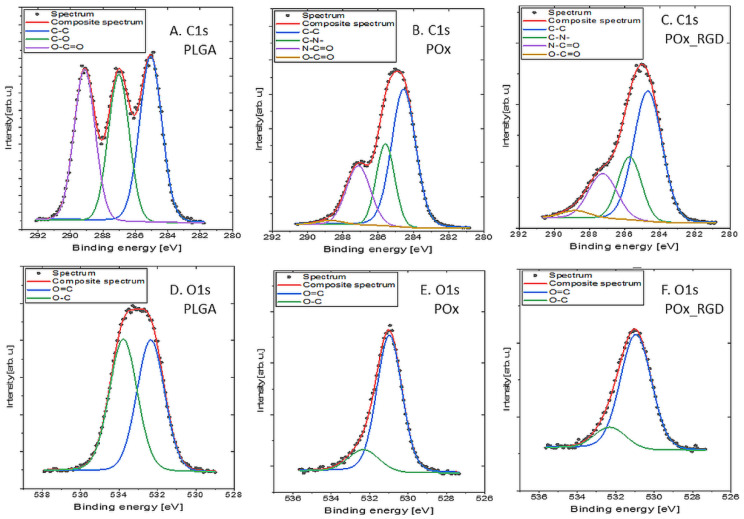
XPS spectra of ingredients: (**A**,**D**) PLGA, (**B**,**E**) POx, and (**C**,**F**) POx_RGD used in manufacturing of PLGA_POx and PLGA_POx_RGD membranes; (**A**–**C**) C_1s_ region and (**D**–**F**) O_1s_ region.

Figure 4 shows the C_1s_ and O_1s_ detailed spectra of PLGA, POx, and POx_RGD, while Table 3 shows the surface composition (at. %) determined by fitting the XPS data.

The C_1s_ spectrum of PLGA (Figure 4A) was fitted with three components: 284.8 eV from C–C and C–H bonds, 286.8 eV from C–O bonds, and 289.0 eV from O–C=O bonds [45]. The spectrum collected in the O_1s_ region (Figure 4D) was fitted with two components: the first one centered at 531.4 eV, attributed to C=O bonds, and the second one at 532.7 eV, attributed to C–O and/or –OH type bonds [45,46,47]. The measured atomic percentages of carbon and oxygen functionalities were very close to the theoretical value for PLGA with this particular ratio of l-lactide to glycolide (85:15), i.e., C = 59 at. % and O = 41 at. %, as shown in our previous study [48].

The C_1s_ spectra of POx (Figure 4B) and POx_RGD (Figure 4C) were fitted with four components: first at 284.8 eV originating from C–C and C–H bonds, second at 285.9 eV from C–O and/or C–N, third at 287.4 eV coming from N–C=O and O–C=O type groups, and lastly at 289.0 eV related to O–C=O type bonds [45,46]. The spectra in the O_1s_ region consisted predominantly of double-bonded oxygen (Figure 4E,F). The spectra collected in the N_1s_ region (data not presented) were fitted with a single line with the binding energy of 399.5 eV, which indicates the presence of either –NH– or N–C=O bonds [45]. The modification of POx with RGD had a faint impact on the chemical composition of the sample, as compared to pristine POx, and this manifested as a decrease in total nitrogen concentration by 1 at. % and an increase in oxygen concentration by 1.4% (Table 3). This was the expected effect, as the theoretical content of RGD in POx_RGD is only 4% of the total molar composition, provided that all piperidine rings in the POx molecules are coupled with the RGD-6-aminocaproic acid derivative.

Moreover, for the POx_RGD sample, a signal from sulfur and silicon was detected (Table 3). The corresponding S_2p_ spectrum was fitted with a doublet structure (doublet separation p_3/2_–p_1/2_ equals 1.18 eV) with the main 2p_3/2_ line centered at 167.7 eV, which indicates the presence of SO_3_^2−^ ions [49]. The Si_2p_ spectrum showed one doublet structure (doublet separation p_3/2_–p_1/2_ equals 0.6 eV) with the main 2p_3/2_ line centered at 101.6 eV, which indicates the presence of C–Si–O type compounds, similar to poly(dimethylsiloxane), for example [50,51]. Such silicon compounds are common sources of contamination, and are often detected in materials analyzed by XPS [52].

### 3.2. Membrane Microstructure

**Figure 5:**
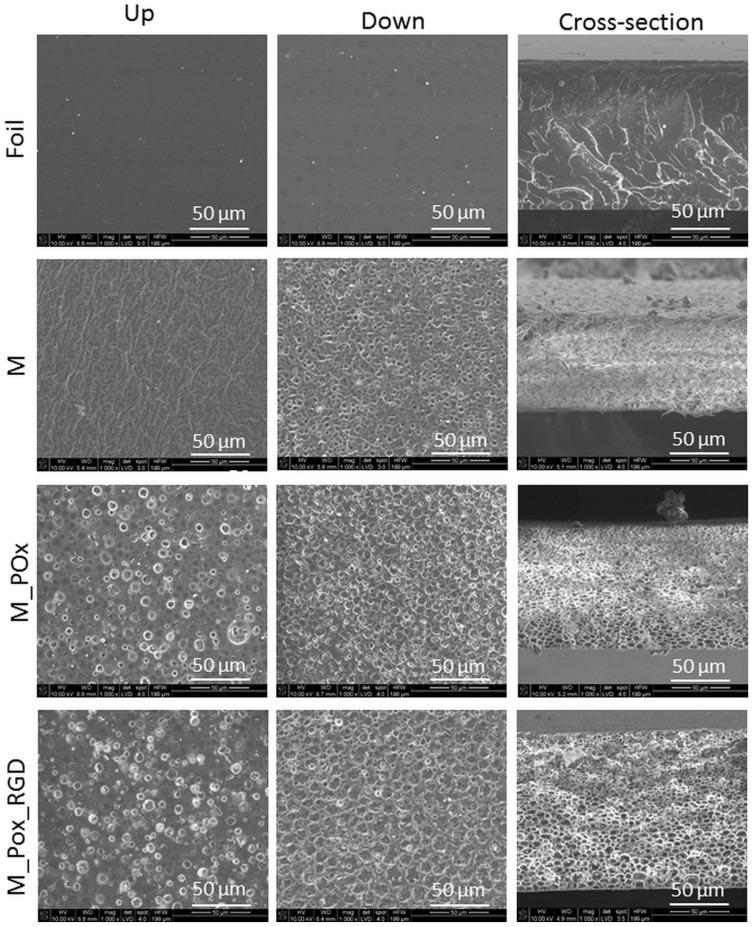
SEM microphotographs of PLGA foil, PLGA membrane (M), PLGA_POx membrane (M_POx), and PLGA POx_RGD membrane (M_POx_RGD); (**up**) surfaces in contact with air and (**down**) in a glass Petri dish during preparation, and the cross-sections produced by breaking after freezing in liquid nitrogen. Magnification ×1000, scale bar = 50 µm.

The microstructures of the reference PLGA foil and the membranes (M, M_POx, M_POx_RGD) as investigated by SEM are shown in Figure 5. It was observed that the air-cured (up) and glass Petri dish-cured (down) surfaces of the foil are smooth, and the picture of the cross-section shows that the foil inside is homogenous and nonporous.

The PLGA membrane (M) was non-porous on the air-cured surface and porous on the glass-cured surface; thus, it exhibited graded porosity at the cross-section, as required for the GTR technique. In our previous paper, we explained the mechanism of such asymmetric membrane formation and the control of their microstructure [12]. This depends on four phenomena: (1) spontaneous phase separation as a consequence of higher PLGA solubility in methylene chloride compared to PEG; (2) inhomogeneous evaporation of the solvent from PLGA and PEG; (3) coalescence PEG-rich domains; and (4) their sedimentation in the course of drying.

M_POx and M_POx_RGD also had an asymmetric microstructure, as shown in the pictures of the cross-sections; however, their air-cured surface also exhibited some porosity. This means that the presence of POx or POx_RGD in the system influences the phase separation between PLGA and PEG, resulting in an altered microstructure after PEG leaching.

### 3.3. Membrane Surface Chemistry

The results of the surface chemistry, wettability, and in vitro tests reported further in this article were performed for the lower surfaces, i.e., those in contact with glass during the preparation of the samples, which were more porous and had more developed surface area, because our aim was to test the membranes in contact with bone cells to confirm their osteogenic potential.

#### 3.3.1. XPS Results

**Figure 6:**
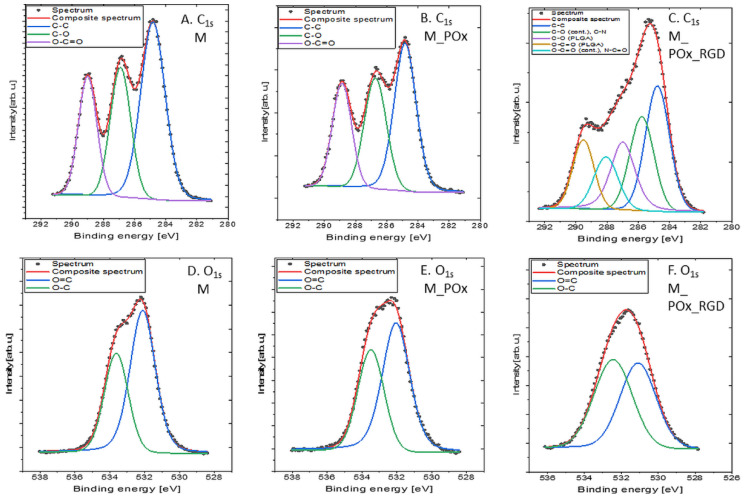
XPS spectra of: PLGA membrane (**A**,**D**), membrane modified with POx (**B**,**E**), and membrane modified with POx_RGD (**C**,**F**); (**A**–**C**) C_1s_ region and (**D**–**F**) O_1s_ region.

The XPS C_1s_ (Figure 6A,B) and O_1s_ (Figure 6D,E) spectra of the PLGA membrane (M) and the membrane modified with POx (M_POx) were decomposed into the same components, as in the case of PLGA: 284.8 eV from C–(C,H) bonds, 286.8 eV from C–O bonds, and 289.0 eV from O–C=O bonds in the C_1s_ region [46], as well as 531.4 eV from C=O bonds and 532.7 eV from C–O and/or –OH type bonds in the O_1s_ region [42,43,44]. The measured concentration of the C–(C,H) component in the M sample was increased and the concentration of C–O bonds was reduced as compared to the PLGA polymer (Table 3). This might be explained by surface contamination from the atmospheric hydrocarbon-containing molecules on the surfaces prepared in laboratory conditions prior to their introduction into the ultra-high vacuum XPS chamber. As shown in other studies, one to several monolayers of contaminants may be present on these surfaces, resulting in an increase in C–(C,H) component concentration [53]. In this sample, silicon traces originating from C–Si–O-type contamination compounds were also detected (Table 3).

In M_POx, apart from the functionalities described above, nitrogen in the form of –NH– or N–C=O bonds was detected. Although nitrogen concentration was only 1.2 at.%, this is proof of POx immobilization on the PLGA surface. It should be kept in mind that XPS provides a meaningful analysis of a thin surface layer between 1 and 10 nm [46]. Therefore, the electrons are expelled not only from the atoms of the very top layer of the surface, but also from several atomic layers below. In our method of membrane modification, only the extreme surface was modified by POx. This means that the XPS signal may also be collected from the subsurface PLGA layers containing no nitrogen, which may result in a reduction in the mean nitrogen content.

In M_POx_RGD, the C_1s_ spectrum (Figure 6C) was fitted with five components, which were the superposition of three components from PLGA (284.8 eV from C–(C,H) bonds, 286.8 eV from C–O bonds, and 289.0 eV from O–C=O bonds) and four components from POx_RGD (284.8 eV from C–(C,H) bonds, 285.9 eV from C–O and C–N bonds, 287.7 eV from N–C=O bonds, and 289.0 eV from O–C=O bonds). The O_1s_ spectrum (Figure 6F) was fitted with two components of double-bonded oxygen at 531.4 eV and single-bonded oxygen at 532.7 eV. The nitrogen concentration was 1.8 at.%, which is also proof of the immobilization of POx_RGD on the surface of the PLGA membrane.

#### 3.3.2. FTIR-ATR Spectroscopy Results

FTIR-ATR spectroscopy was used to: (1) verify if the entire porogen load was removed from the PLGA/PEG/POx_RGD blend during PEG leaching in water, and (2) to find out if POx_RGD was really immobilized on the surface of PLGA.

**Figure 7:**
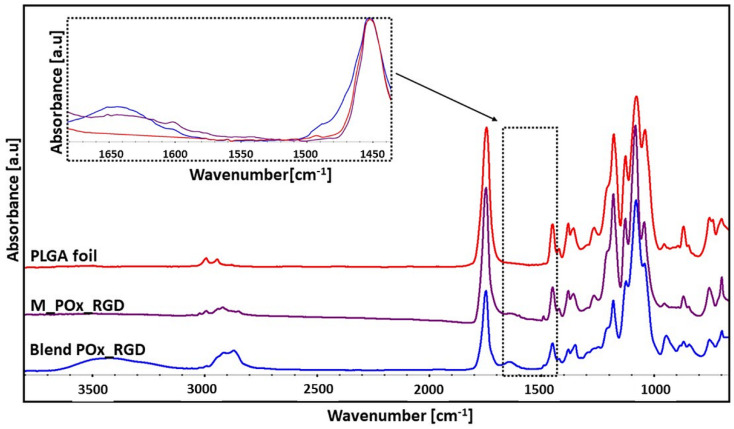
FTIR-ATR spectra of PLGA foil, PLGA POx_RGD membrane, and PLGA POx_RGD blend.

As shown in Figure 7, the FTIR spectrum of the PLGA and PEG blend containing POx_RGD had all the bands characteristic for both main ingredients: PEG (at 3438 cm^−1^ due to the stretching vibrations of the hydroxyl groups, at 2866 cm^−1^ from stretching vibrations of the CH_2_ groups, and at 1092 cm^−1^ attributed to the stretching vibrations of the C–O groups) and PLGA (at 1745 cm^−1^ due to the stretching vibration of the carbonyl groups, bands between 1450 and 1360 cm^−1^ originating from CH bending vibrations, and bands at 1180 and 1080 cm^−1^ from C–O–C stretching vibrations). Moreover, a band at 1620 cm^−1^ from the C=O stretching vibrations, which is characteristic for polyoxazoline structure [36] and POx_RGD (see Figure 3B), was visible. This band was not observed in the spectrum of the PLGA foil, which proves, indirectly, that POx_RGD can be found in the blend. After leaching of PEG, this band was still detected in the M_POx_RGD membrane, although its intensity was reduced. This suggests that POx_RGD is immobilized on the surface of the membranes and PEG is no longer present in the membrane after the leaching procedure. One might argue that the intensity of the band at 1620 cm^−1^ is quite low; however, one must keep in mind that the concentration of POx_RGD is only 1 wt. % in respect to PLGA. Moreover, FTIR-ATR, although regarded as surface-sensitive, collects data from a depth of up to 1 µm [54], i.e., where there is only PLGA, and not only from the external surface, where we expect the presence of POx_RGD. 

#### 3.3.3. Wettability and Surface Free Energy

**Figure 8:**
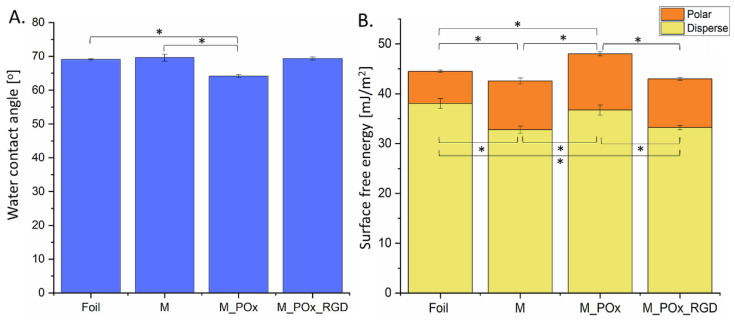
(**A**) Water contact angle and (**B**) surface free energy of PLGA foil (foil), PLGA membrane (M), PLGA_POx membrane (M_POx), and M_POx_RGD membrane. Statistical significance for *p* < 0.05 (*), according to one-way ANOVA with Fisher LSD post-hoc test.

The results of the wettability and surface free energy assays of the PLGA foil, M, M_POx, and M_POx_RGD, are shown in Figure 8A and Figure 8B, respectively. It was apparent that all materials analyzed were moderately hydrophilic, with a water contact angle equal to or below 70° (Figure 8A). Statistical analysis showed that the wettability of M_POx was significantly lower (68.0° ± 0.9°) than that of the foil (68.8° ± 0.3°) or membrane without modification (69.6° ± 1.6°). This suggests that the amphiphilic POx molecules were immobilized on the surface of the PLGA, presumably due to the interaction of hydrophobic 2-butyl-2-oxazoline with hydrophobic PLGA chains and exposed hydrophilic 2-metyl-2-oxazoline chains. Interestingly, on the membrane modified with POx_RGD, the wettability decreased again. This was a surprising result, because RGD sequences are hydrophilic and, in other studies, they usually decrease the water contact angle [52,53,54].

Further, it should be considered that the wettability was measured on porous surfaces for M, M_POx, and M_POx_RGD. According to the Wenzel and Cassie–Baxter theories, as well as the equation developed by Kubiak et al. [55], the values of the measured apparent water contact angle can be strongly affected by the surface roughness. Accordingly, topographical features have an impact on wettability, and only samples with similar surface roughness can be compared directly. The results of the surface free energy (SFE) calculations (Figure 8B) showed that the total SFE as well as its polar part were the highest for M_POx. The presence of RGD resulted in a decrease in the SFE values.

### 3.4. Membrane Biological Properties

**Figure 9:**
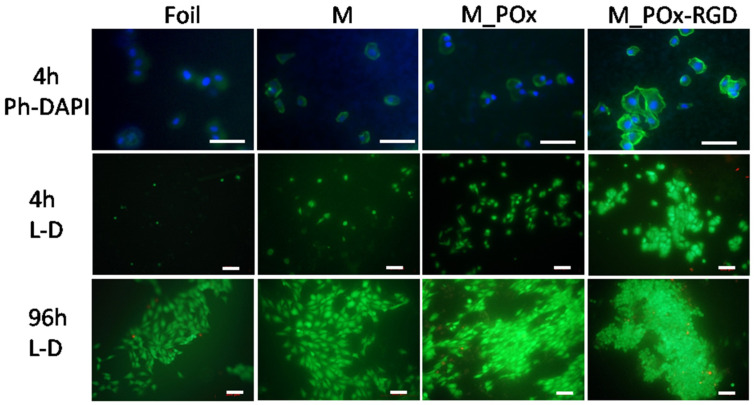
Morphology of MG-63 cells cultured on PLGA foil (Foil), membranes (M), membranes modified with POx (M_POx), and POx_RGD (M_POx_RGD). Cells were stained live/dead and for phalloidin and DAPI. Scale bar = 100 µm.

To verify if the PLGA membranes produced were compatible with bone cells in vitro and if their modification with POx_RGD further improved the surface biological properties, the morphology and viability of MG-63 osteoblast-like cells were studied in direct contact with the membranes. The cells were cultured on the membranes and the reference PLGA foil for 4, 24, and 96 h. The morphology of the cells stained with phalloidin and DAPI to visualize actin fibers and nuclei, respectively (Figure 9, panel 1), showed that 4 h after seeding, the cells seeded on M_POx_RGD were very well-spread and had the best-developed actin fibers as compared to cells cultured on the foils or membranes without modification or modified only with POx.

When live/dead staining was applied (Figure 9, panel 2) it was observed that the majority of the cells in all the samples were alive, whereas the number of dead cells was below 3%. Cell number as well as cell spreading were also the highest on M_POx_RGD. At 96 h after seeding (Figure 9, panel 3), the cells proliferated on all the materials, and the number of cells seemed to be the highest on M_POx_RGD.

**Figure 10:**
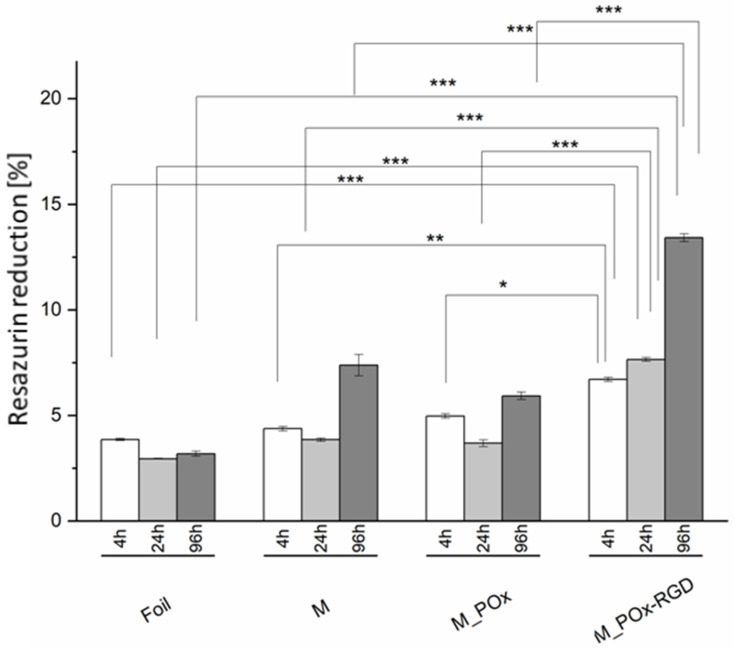
MG-63 cell viability measured by resazurin reduction for PLGA foil (Foil), PLGA membrane (M), PLGA membrane modified with POx (M_POx), and with POx_RGD (M_POx_RGD). * *p* < 0.05, ** *p* < 0.01, *** *p* < 0.001: statistical significance according to one-way ANOVA with Fisher LSD post-hoc test.

The results of the resazurin reduction test (Figure 10) showed that the viability of the cells cultured on M_POx_RGD was the highest for all time points as compared to the other membranes and the reference PLGA foil. It was shown that the cell viability on M_POx_RGD exceeded that of M_POx and M in a statistically significant manner (*p* < 0.001).

All the materials were found to be cytocompatible with osteoblast-like cells; however, M_POx_RGD was found to support cell adhesion, proliferation, and viability to the highest extent. Our results suggest that the presence of RGD may ensure faster colonization of the surface with host cells, thus reducing colonization with pathogenic bacteria according to the phenomenon of the ‘race for the surface’ [56,57].

## 4. Conclusions

In this study, we developed a manufacturing method for porous asymmetric PLGA membranes modified with POx_RGD. The membranes were obtained by phase separation and preferential adsorption of POx_RGD molecules at the PLGA/PEG interface with POx_RGD exposed to hydrophilic PEG, followed by solvent evaporation and PEG leaching.

Prior to membrane manufacturing, all the ingredients used in production, i.e., PLGA, PEG, POx, RGD, and POx_RGD, were tested by using Raman, FTIR-ATR, and XPS spectroscopic techniques. Detailed analysis of the spectra confirmed that RGD was successfully coupled with POx.

The membranes had an asymmetric microstructure, as shown in the SEM pictures of both the surfaces and cross-sections; the glass-cured surface was more porous and was characterized by a higher surface area as compared to the air-cured surface.

The XPS and FTIR-ATR studies confirmed that POx_RGD was immobilized on the membrane surface; however, this modification practically did not influence the surface wettability and surface free energy values.

The in vitro tests showed that the POx_RGD-modified PLGA membranes supported osteoblast-like cell adhesion, proliferation, and viability to the highest extent, as compared to the other control membranes or PLGA foil.

In summary, the one-step phase separation process between PLGA, PEG, and POx_RGD dissolved in DCM, followed by drying and leaching of PEG, resulted in asymmetric PLGA membranes with enhanced biological properties, which might be considered for the guided tissue regeneration technique in periodontology and bone tissue engineering.

## Figures and Tables

**Table 3:** Surface composition (at. %) determined by fitting XPS data of ingredients used to produce the membranes (PLGA, POx and POx_RGD) as well as PLGA membranes: non-modified (M), and modified with POx (M_POx) and POx_RGD (M_POx_RGD).

Element	C					N	O		Si	S
Energy [eV]	284.8	285.9	286.9	287.7	289.0	399.5	531.4	532.7	101.6	167.7
Chemical shift	C–C C–H	C–O C–N (POx)	C–O (PLGA)	O–C=O N–C=O (POx)	O–C=O	–NH– N–C=O	O=C O–Si	O–C	Si–O	SO_3_^2−^
Ingredients										
PLGA	24.0	^bdl^	19.1	^bdl^	20.0	^bdl^	18.2	18.8	^bdl^	^bdl^
POx	39.9	17.0	^bdl^	16.1	1.0	14.0	10.1	1.9	^bdl^	^bdl^
POx_RGD	40.6	15.6	^bdl^	13.5	2.2	13.0	11.4	2.0	1.1	0.7
Membranes										
M	30.6	^bdl^	16.4	^bdl^	16.0	^bdl^	21.7	13.1	2.2	^bdl^
M_POx	27.3	^bdl^	20.8	^bdl^	14.9	1.2	21.3	14.0	0.5	^bdl^
M_POx_RGD	20.3	14.9	12.7	8.3	9.6	1.8	14.9	15.8	1.7	^bdl^

^bdl^—below detection limit.

## Data Availability

Not applicable.
